# The Limits of Agency: Young Children’s Memory May Not Benefit From Choice

**DOI:** 10.1525/collabra.137316

**Published:** 2025-05-30

**Authors:** Naoya Tani, Ingrid R. Olson, Nora S. Newcombe

**Affiliations:** 1 Department of Psychology and Neuroscience, Temple University, Philadelphia, PA, USA

**Keywords:** memory, agency, learning, cognitive development

## Abstract

It is commonly claimed that curiosity, agency, and choice enhance learning and memory in children. However, the few studies that have investigated this in young children reveal mixed effects on memory. To test this, in Experiment 1, children aged 4–7 years watched short cartoon clips and then viewed one of two endings: either in an “active” condition, where they made choices about which ending to view, or a “yoked” condition, where choices were made for them. A surprise memory test conducted 6–8 days later showed no significant difference between conditions in either recognition or binding tasks. In Experiment 2, a within-subject design was employed to control for individual differences. Again, no significant differences were found between conditions. Bayes factor analyses provided evidence supporting the null hypothesis in this child-friendly, cartoon-based paradigm. While our findings suggest that, under these specific task conditions, the agency does not enhance memory in 4- to 7-year-olds, further research is needed to clarify whether different task structures, feedback, or age groups might reveal more robust effects. Potential boundary conditions and developmental implications are discussed.

Children are often seen as full of curiosity, a trait that drives many exploratory behaviors, propelling children to engage with their environment in ways that foster learning and cognitive development. Exploration involves making choices that allow children to gather information and learn from their surroundings (Repacholi & Gopnik, 1997). This process of exploration is a purposeful activity aimed at making sense of the world. In educational settings, the concept of agency—where learners have control over their actions and decisions—has been claimed to play a critical role in enhancing learning effectiveness (Gureckis & Markant, 2012).

In adults, some research has shown that having control over decisions—known as the agency effect—enhances memory. Studies indicate that when adults make choices during learning tasks, it improves memory retention, particularly in recognition tasks (Ding et al., 2021; Murty et al., 2015). This effect likely occurs because making choices can heighten attention and prompt more elaborative encoding, thereby leading to improved memory encoding and retrieval. Even inconsequential choices can enhance declarative memory, suggesting that the act of choosing plays a significant role in how information is encoded and retrieved. However, other studies find that choice-making only benefits memory under certain conditions, such as shorter delays between action and outcome (Hon & Yeo, 2021) or in individuals with better cognitive abilities (Markant, 2020). There is also evidence that the impact of agency on memory may depend on task-specific context (Tsuji & Imaizumi, 2022).

Mixed findings also characterize the smaller literature on children. Katzman and Hartley (2020) observed that the mnemonic benefit of choice was evident only when the choices had high utility—participants remembered information better when their choices led to higher rewards. The “high utility effect” of choice was consistent across participants aged 8 to 25. Using a different paradigm, Ruggeri et al. (2019) found that choice improved memory for visual information but not spatial information in children aged 5 to 11. Notably, memory enhancement from choice was more pronounced in older children, particularly beginning around 8 years old, suggesting that the effects of choice strengthen over the school years. Similarly, McComas et al. (1997) found no significant benefit from choice-making in 6 and 7-year-olds.

To provide a broader overview of how choice-making influences memory performance across various age ranges, tasks, and contexts, we summarized several representative studies in Table 1.

The agency effect may be less pronounced or even absent in younger children, possibly due to developmental factors such as emerging cognitive control and metacognitive abilities (Luna et al., 2015; Rosenbaum & Hartley, 2019). Brod (2021) suggests that the effectiveness of active learning strategies, including agency and choice-making, may vary with learners’ developmental stages. Younger children might not benefit from these practices as much as older individuals due to differences in cognitive capacities, prior knowledge, and metacognitive abilities. This developmental perspective implies that what is effective for older learners may not apply to younger children. However, despite the importance of understanding how agency affects learning in early childhood, there is a paucity of research on children under the age of 8. A few recent studies in the word-learning research (Ackermann et al., 2020; Bothe et al., 2024; Sivridag & Mani, 2024) illustrate how active versus passive learning can yield inconsistent results in younger populations, highlighting that choice does not uniformly enhance learning outside of older age ranges. This underlines the need for further investigation into whether and how agency benefits memory, specifically in children under age 8.

## Current Study

Our study aims to contribute to this body of research by focusing on younger children, including 4-year-olds, and exploring how choice-making affects their memory. We designed an environment where children could naturally influence the narrative by making meaningful choices within a cartoon story. By offering only two choices per task, each with significant consequences for the continuation of the story, we sought to engage children in a way that felt natural and immersive.

We hypothesized that children in the active/agency condition would exhibit significantly higher recognition and binding scores than those in the yoked condition. These hypotheses were based on evidence from older children and adults demonstrating that choice-making can enhance declarative memory (e.g., Murty et al., 2015; Ruggeri et al., 2019).

The use of popular cartoons was intended to capture the children’s attention and maintain their interest throughout the tasks. Conducting the study online allowed children to participate from the comfort of their homes, reducing potential stress or distractions that might occur in unfamiliar environments. Through this approach, we aim to provide a deeper understanding of how choice-making influences memory in young children, particularly in situations where the choices are simple yet meaningful.

## Experiment 1

### Methods

#### Participants

We recruited 64 children (39 females, mean age = 5.87 years) using a combination of previous lab participants, family referrals, and Facebook advertisements. We aimed to recruit approximately 60 participants in Experiment 1, guided by sample sizes in previous studies. We reasoned that if choice-making had a moderate effect, we would have sufficient power (i.e., 0.80) to detect it. Initial screening using an online form looked at the inclusion and exclusion criteria. Inclusion criteria were that children be typically developing, between the ages of 4 and 7 years, and English-speaking. We excluded children with cognitive or language impairments, developmental disorders, significant hearing or visual impairments, or born before 32 weeks of gestation. Once eligibility was confirmed, families were asked to participate in the study. Of the initial 64 participants, 7 were excluded: 6 children did not return for the memory test (session 2) within the required 6–8 day timeframe, and 1 child was identified as a duplicate participant. The final dataset included 57 children (36 females, mean age = 5.82 years). Participants were randomly assigned to either the active condition (29 participants, 19 females, mean age = 5.96 years) or the yoked condition (28 participants, 18 females, mean age = 5.68 years). Participants received a $20 Amazon gift card after completing the encoding task (session 1) and a $30 Amazon gift card after completing the memory test (session 2). Table 2 shows the demographics in Experiment 1.

#### Materials and Procedure

##### Session 1 (Encoding).

Session 1 was conducted online via Zoom. Sessions took about 40–45 minutes, including the introduction (about 10 minutes) and the encoding task (about 30–35 minutes). Each participant was randomly assigned to one of the two conditions: active or yoked. The task was conducted on participants’ computers using Qualtrics, and lab personnel continuously monitored the sessions.

At the beginning of session 1, the task was introduced with the kid-friendly language, and a practice game was conducted using a clip from the cartoon *Masha and the Bear*. The practice game was tailored to the participant’s assigned condition (active or yoked). Participants in the active condition were prompted to make a choice after a short cartoon clip of *Masha and the Bear*, while for the participants in the yoked condition, the choice was made automatically after the same short clip. Specifically, in the practice of yoked condition, the narration explicitly indicated that the experimenter (or the character) would make the choice for them, for example: “Uhm… I pick popcorn!”. This practice ensured that children in both conditions understood the procedure and felt comfortable participating before moving on to the main experiment.

**Figure 1.**
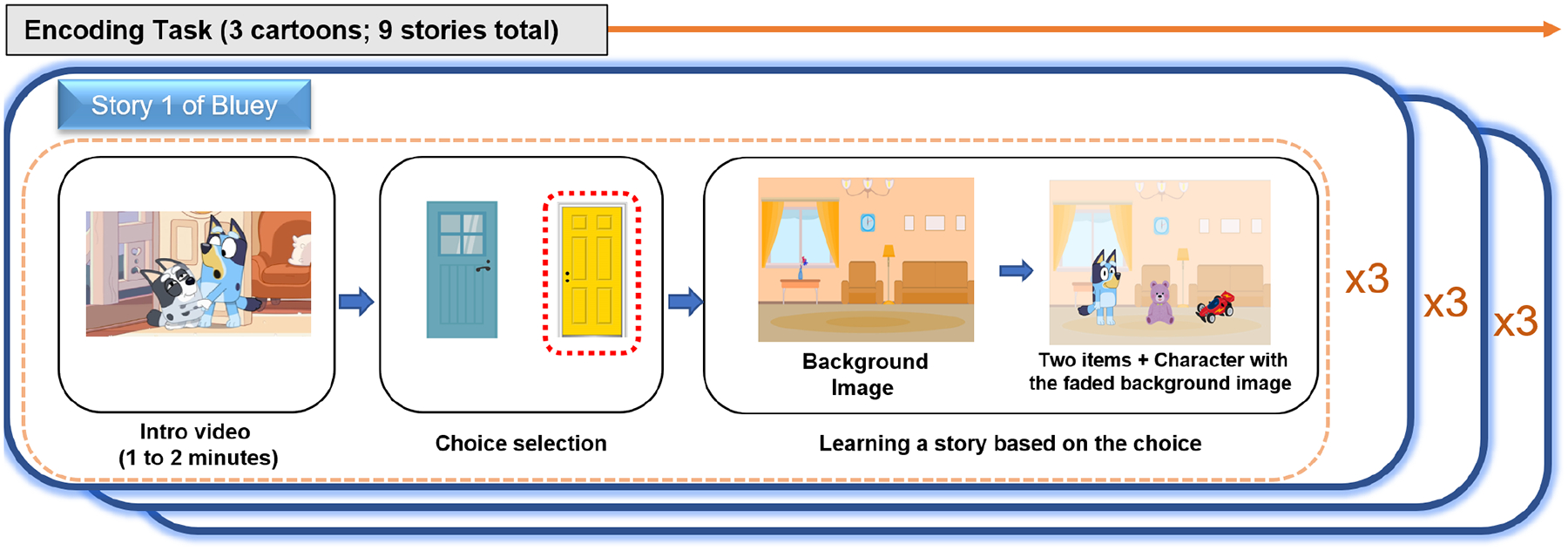
Overview of the encoding task used in Experiment 1. Each participant watched nine stories, with each story including an introduction video (1 to 2 minutes), a choice selection phase, and a learning phase involving a background image and two items with the character in a faded background image. The stories were derived from three cartoons: *Bluey, Peppa Pig*, and *Paw Patrol*. During the choice selection phase, participants in the active condition were asked to choose which story continuation they wanted to see, whereas in the yoked condition, the outcome was automatically selected without the child’s input.

The encoding task comprised short cartoon segments from *Bluey*, *Peppa Pig*, and *Paw Patrol*, with each series contributing three unique stories (total of nine unique and independent stories). As shown in Figure 1, each story followed a **three-step flow**:

1. **Introduction Clip (1–2 minutes)** – An original excerpt from the cartoon.
2. **Choice Selection** – Children either actively decided which path to follow (*active condition*) or observed a prerecorded “decision” made by the character (*yoked condition*).
3. **Post-Cartoon Story** – A custom narrative continuation reflecting the chosen path.

In the **active** condition, selections (e.g., “blue door” vs. “yellow door”) determined which scene and items would appear. For example, choosing the “blue door” led to an outdoor setting with specific props, whereas choosing the “yellow door” led indoors with a different set of objects. By contrast, in the **yoked** condition, the program automatically selected one option (e.g., “Bluey decides to choose the yellow door!”) almost immediately after presenting it on screen. This phrasing gave the impression that the character was making an autonomous decision, which children typically accepted without question.

To ensure that children’s choices felt meaningful, each story featured two distinct sets of items and backgrounds that would appear depending on which option (e.g., “blue door” or “yellow door”) was selected. We did not explicitly tell children that different items or backgrounds would result from their choice, only that their selection would continue the story. This allowed them to discover that their decision changed what they saw in the subsequent clip (e.g., outdoor props versus indoor toys). In the yoked condition, a prerecorded selection automatically led to one of the same two options, maintaining identical outcomes but removing the child’s personal control.

We specifically designed the task so that children would step into the story world, helping or making decisions on behalf of the cartoon characters. Rather than positioning the child as an external observer who merely “presses a button,” we framed each choice as assisting the character (e.g., “You pick one for Bluey!”). This approach aimed to foster a child-friendly, immersive experience, in which children felt they were actively guiding the narrative. Although this design may have reduced explicit statements linking the child’s action to the subsequent outcome (“You chose X, so Y happened”), it helped maintain the story’s internal consistency and encouraged deeper engagement with the characters.

To illustrate, one *Bluey* story featured Bluey’s cousin, Muffin, who was tired and prone to tantrums. After the brief intro clip, the narrator said, “Bluey wants to play, but she can’t decide where to go!” In the active condition, the child was prompted, “You pick one for Bluey!”; in the yoked condition, a prerecorded narration made the choice for them. Following that choice, the story advanced to a custom scene (e.g., “Bluey opens the yellow door and enters the living room, where she finds a purple teddy bear and a red toy car.”).

This sequence was repeated for all nine stories in a pseudorandom order. To standardize experiences in the yoked condition, we used a predetermined sequence of “choices,” so that all participants in this condition saw the same outcomes as if the character had decided independently. Both the child’s actual selections (active condition) and the automated choices (yoked condition) were recorded electronically via Qualtrics.

##### Session 2 (Surprise Memory Test).

The surprise memory test was conducted 6 to 8 days later, also online via Zoom. Similar to session 1, session 2 was conducted on participants’ computers using Qualtrics, and lab personnel continuously monitored the session. This session was about 30–40 minutes, including the number game, which took less than 15 minutes, before the memory task, which required about 15 to 25 minutes. For the number game, participants completed the Woodcock-Johnson IV Tests of Early Cognitive and Academic Development (ECAD) Number Sense subtest (Schrank & Wendling, 2018). This test is not relevant to this study, however. Children were not informed that a memory test would occur; they were only told that they would play another game in 6–8 days. The memory test consisted of 54 questions divided into two types: four-alternative forced choice (4AFC) recognition memory questions and three-alternative forced choice (3AFC) character-source questions. There were 27 recognition memory questions (9 for background images, 18 for item images). Each recognition memory question included four options:

- Correct: The exact image seen during the post-cartoon story from Session 1
- Lure: A similar image to the correct one (e.g., if the correct image is a purple teddy bear, the lure might be another teddy bear with a different color),
- Foil 1 and Foil 2: Both of these images were not shown in Session 1 and were generally more distinct from the correct item. However, having two foils helps ensure that children do not rely on the mere process of elimination (e.g., “Only one image looks like a teddy bear, so that must be it”). In most cases, Foil 1 and Foil 2 were also similar to each other (e.g., two different plush animals) but notably different from the correct item.

**Figure 2.**
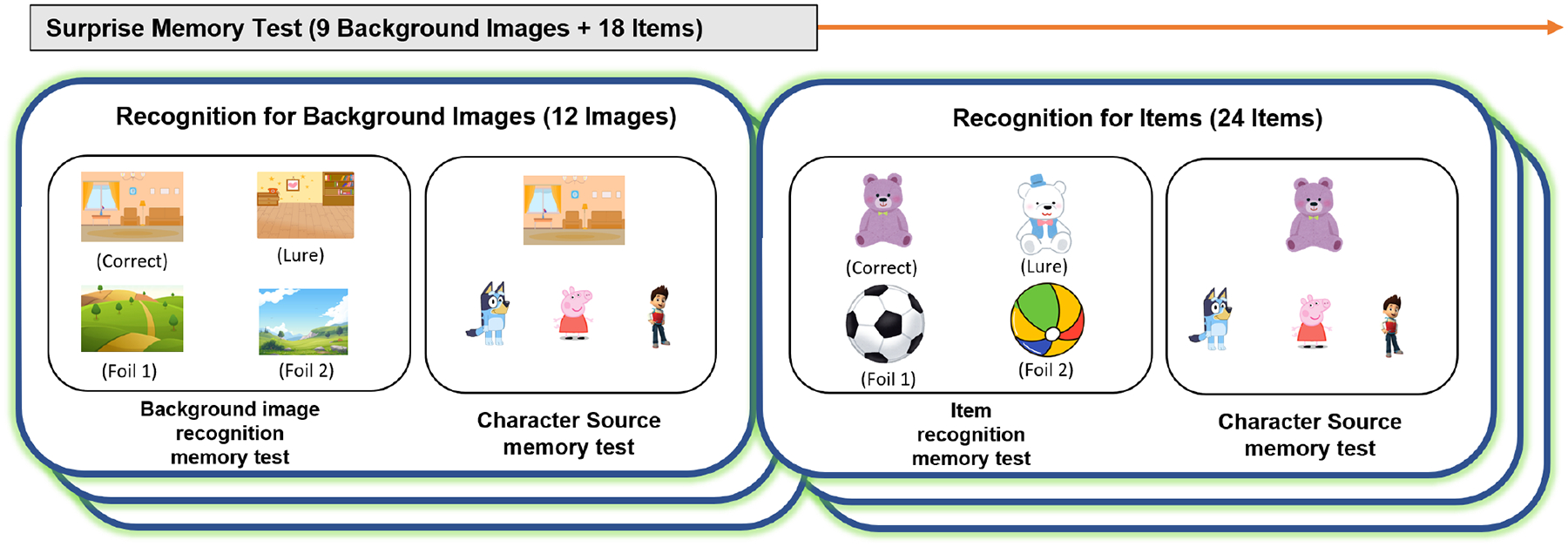
Overview of the surprise memory test used in Experiment 1. The test consisted of recognition memory questions for background images (9 images) and items (18 items), followed by character-source memory tests. The recognition memory test for background images involved four options: correct, lure, foil 1, and foil 2. The recognition memory test for items also involved four options: correct, lure, foil 1, and foil 2. The character-source memory test asked participants to identify which character was associated with the background image or item, using three options: Bluey from *Bluey*, Peppa from *Peppa Pig*, and Ryder from *Paw Patrol*.

There were also 27 character-source questions asking which character was associated with the background and item images participants selected in the recognition memory task. Each character-source question was asked after each recognition memory question. These questions used a 3AFC format with the main characters from each cartoon (Bluey from *Bluey*, Peppa from *Peppa Pig*, and Ryder from *Paw Patrol*) as options (see Fig. 2). The child’s responses in the memory test were recorded electronically through Qualtrics.

#### Data Analysis

All statistical analyses were performed in R version 4.4.0 (R Core Team, 2024) and R Studio version 2024.04.1 (RStudio Team, 2020). Data were analyzed to compare performance between the active and yoked conditions across different types of memory measures. Recognition memory for item images and recognition memory for background images were analyzed separately. For recognition memory, correct responses were scored as 1, and incorrect responses were scored as 0. *t*-tests were conducted to compare the active and yoked conditions for both recognition memory for item images and recognition memory for background images.

Similarly, character-source memory for item images and character-source memory for background images were analyzed separately. For character-source memory, a correct recognition memory response (scored as 1) was required for the subsequent character-source question to be considered. Only if the participant correctly identified the background or item, and subsequently the correct character associated with it, was it scored as a correct response for character-source memory. If recognition was 0 or if recognition was 1 but the character choice was incorrect, it was scored as 0. The independent samples *t*-tests were conducted to compare the active and yoked conditions for recognition and character-source memory. Bonferroni correction was applied to account for multiple comparisons, ensuring that the statistical significance threshold was appropriately adjusted.

Bayes factor analyses using the Jeffreys–Zellner–Siow (JZS) default prior were also conducted in BayesFactor package (Morey & Rouder, 2024) in R to assess the strength of evidence for the null hypothesis, indicating whether the data provided moderate to strong support for the lack of an effect of agency on memory performance in young children. The JZS prior is relatively noninformative, suitable for detecting moderate-to-large effect sizes (Rouder et al., 2009). Consequently, if our study were underpowered to detect a moderate effect of agency, we would expect inconclusive Bayes factors around 1.0. Instead, we found moderate-to-strong support for the null, suggesting that any memory benefit from agency, if present, is likely small or absent in this population under our task conditions.

## Experiment 2

Experiment 2 was designed as a within-subject study, in contrast to the between-subjects design used in Experiment 1, to build upon the findings with several key modifications aimed at controlling for individual differences and enhancing the experimental design. The primary differences between Experiment 1 and Experiment 2 are described below.

### Methods

#### Participants

We recruited 40 children (20 females, mean age = 6.06 years) through previous lab participants, family referrals, and Facebook advertisements. Participants who participated in experiment 1 were not allowed to participate in this experiment 2. Exclusion criteria and inclusion criteria remained the same as in Experiment 1. Of the initial 40 participants, 3 were excluded: 2 children had technical issues with the task during session 2 (i.e., the audio clips during the memory task did not properly work), and 1 child was found to have a neurodevelopmental disorder after session 2. This resulted in a final dataset of 37 children (20 females, mean age = 6.07 years). Table 2 shows the distribution of demographics in Experiment 2.

#### Materials and Procedure

Experiment 2 utilized four cartoons instead of three, incorporating *Pokémon* in addition to *Bluey, Peppa Pig*, and *Paw Patrol*. This resulted in a total of 12 stories, with each cartoon contributing three stories. The overall structure of the stories remained the same as Experiment 1, consisting of an introduction cartoon clip, a choice phase, and a subsequent narrative continuation. However, the number of stimuli and memory questions increased correspondingly.

##### Session 1 (Encoding).

The encoding session was conducted online via Zoom and was designed as a within-subject study. Each participant experienced both the active and yoked conditions. Children watched and listened to a series of 12 stories. The introduction cartoon clips followed a choice phase and a narrative continuation. The choice phase and narrative continuation were created by lab personnel using pre-prepared materials, consistent with the procedures in Experiment 1.

A practice game was conducted before each condition using a clip from the cartoon *Masha and the Bear*, the same as Experiment 1. The practice game was tailored to the participant’s first condition (active or yoked). Before the second condition began, the participants were given an explanation of the differences from the first condition. The order of agency conditions was counterbalanced across the participants.

For the yoked condition only, the choice prompt narration was shortened to prevent participants from engaging in the choice-making activity. For example, in the Bluey story, the prompt was modified to “Bluey wants to play. One is the blue door that will take her outside. The other is the yellow door that will keep her inside.” This change was only implemented in the yoked condition.

##### Session 2 (Surprise Memory Test).

The surprise memory test was conducted 6–8 days later, also online via Zoom. This session was about 40–60 minutes, including The Kaufman Brief Intelligence Test (2nd ed.) Revised (KBIT–2 Revised; Kaufman & Kaufman, 2022), which took about 20 to 30 minutes in order to measure a child’s IQ intelligence quotient, and the memory test, which took about 20 to 30 minutes. The memory test in Experiment 2 consisted of 72 questions divided into two types: recognition memory questions (4AFC) and character-source questions (4AFC). There were 36 recognition memory questions (12 for background and 24 for item images). Each recognition memory question included four options: correct (the image seen in the post-cartoon story from session 1), lure (similar to the correct image but not seen in session 1), foil (not seen in session 1), and foil 2 (similar to foil 1 but not seen in session 1).

There were also 36 character-source questions asking which character was associated with the background and item images participants selected in the recognition memory task. These questions used a 4AFC format, in contrast to a 3AFC format in Experiment 1, with the main characters from each cartoon (Bluey from *Bluey*, Peppa from *Peppa Pig*, Ryder from *Paw Patrol*, and Ash from *Pokémon*) as options.

#### Data Analysis

Data analysis procedures were consistent with those used in Experiment 1. Recognition memory for item images and recognition memory for background images were analyzed separately using *t*-tests to compare the active and yoked conditions. Similarly, character-source memory for item images and character-source memory for background images were analyzed separately. For each type of memory, the mean scores were calculated for both conditions. The paired-sample *t*-tests were conducted to compare the active and yoked conditions for each type of memory measure. Bayes factor analyses were also conducted to assess the strength of evidence for the null hypothesis, indicating whether the data provided moderate to strong support for the lack of an effect of agency on memory performance in young children. Interpretation of Bayes factors was based on the guidelines by Jeffreys (1961) and Lee & Wagenmakers (2012).

## Results

### Experiment 1

#### Recognition Memory for Background Images

Recognition memory for background images was compared using a Welch Two Sample *t*-test. The results indicated no significant difference between the active and yoked conditions. The mean score for the active condition (*M* = 0.690, *SD* = 0.310) was not significantly different from the yoked condition (*M* = 0.643, *SD* = 0.353), *t*(53.5) = 0.530, *p* = .594, 95% CI [−0.130, 0.222] (*see* Fig. 3a, *left side*). Bayes factor provided moderate evidence for the null hypothesis (*BF*_10_ = 0.302, ±0.01%), supporting the lack of an agency effect on recognition memory for background images.

#### Recognition Memory for Item Images

Recognition memory for item images also showed no significant difference between the active and yoked conditions. The mean score for the active condition (*M* = 0.567, *SD* = 0.197) was not significantly different from the yoked condition (*M* = 0.593, *SD* = 0.236), *t*(52.5) = −0.454, *p* = .651, 95% CI [−0.142, 0.089] (*see* Fig. 3a, *right side*). Bayes factor provided moderate evidence for the null hypothesis (*BF*_10_ = 0.292, ±0.01%), indicating no significant agency effect on recognition memory for item images.

#### Character-Source Memory for Background Images

Character-source memory for background images showed no significant difference between the active and yoked conditions. The mean score for the active condition (*M* = 0.460, *SD* = 0.265) was not significantly different from the yoked condition (*M* = 0.484, *SD* = 0.248), *t*(54.9) = −0.358, *p* = .721, 95% CI [−0.161, 0.112] (*see* Fig. 3b, *left side*). Bayes factor provided moderate evidence for the null hypothesis (*BF*_10_ = 0.283, ±0.01%), suggesting no significant agency effect on character-source memory for background images.

#### Character-Source Memory for Item Images

Character-source memory for item images also showed no significant difference between the active and yoked conditions. The mean score for the active condition (*M* = 0.351, *SD* = 0.201) was not significantly different from the yoked condition (*M* = 0.405, SD = 0.236), *t*(53.1) = −0.932, *p* = .356, 95% CI [−0.171, 0.062] (see Fig. 3b, right side). Bayes factor provided anecdotal evidence for the null hypothesis (*BF*_10_= 0.386, ±0.01%), indicating limited but still supportive evidence for the lack of an agency effect on character-source memory for item images.

### Experiment 2

#### Recognition Memory for Background Images

**Figure 4.**
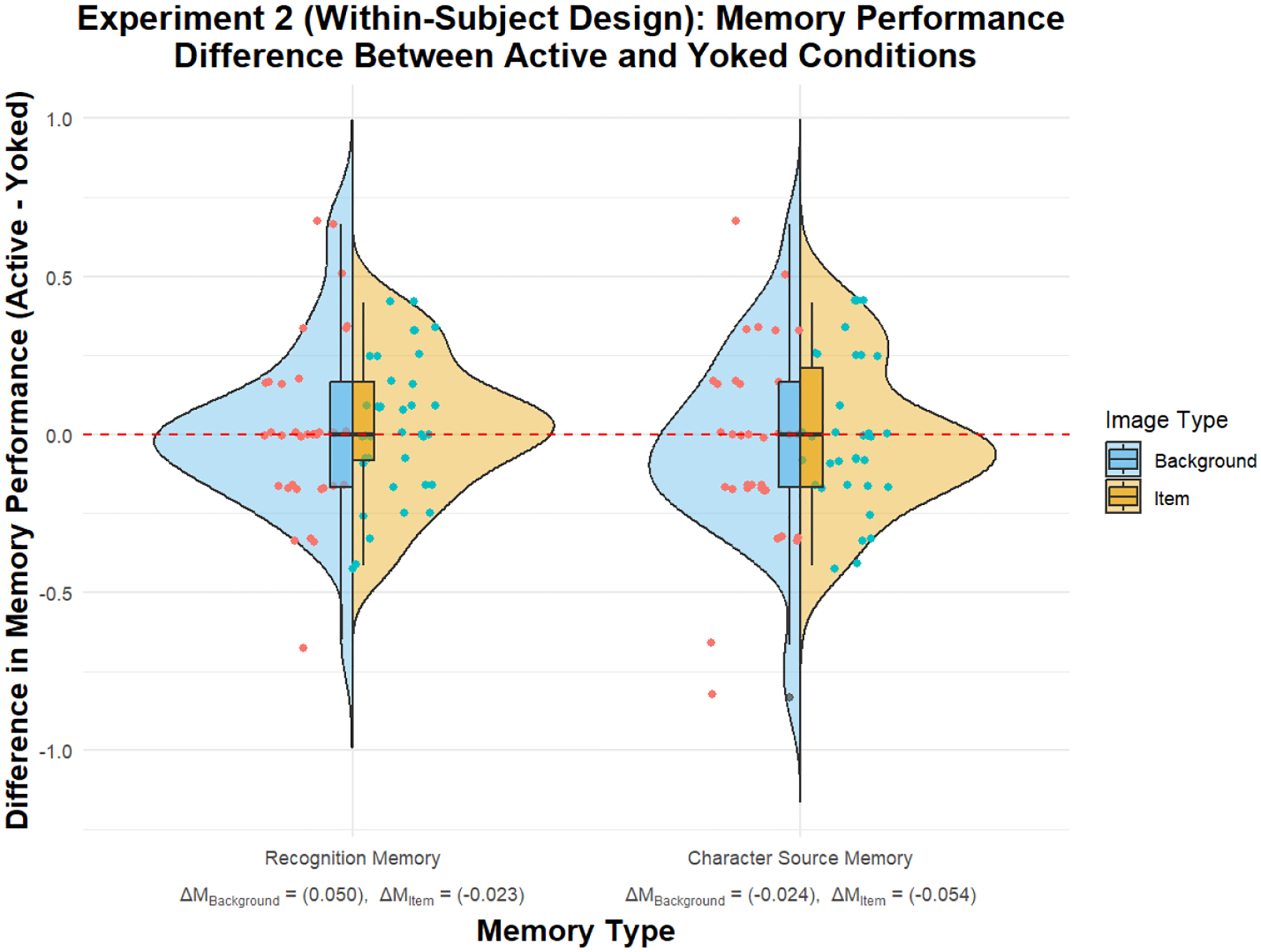
Within-participant comparison of memory performance differences between active and yoked conditions in Experiment 2, for both recognition memory and character-source memory. The split-violin plot shows the distribution of differences in memory performance (Active - Yoked) across the two memory types, with background and item images distinguished by color. The left side of each violin represents recognition memory, while the right side represents character-source memory. The points within the violins indicate individual data points, and the overlaid boxplots represent the interquartile range and median of the differences. The red dashed line indicates a zero difference, representing no change between conditions.

Recognition memory for background images was analyzed using a paired *t*-test, showing no significant difference between the active and yoked conditions. The mean score for the active condition (*M* = 0.690, *SD* = 0.320) was not significantly different from the yoked condition (*M* = 0.640, *SD* = 0.304), *t*(147) = 0.492, *p* = .624, 95% CI [−0.041, 0.068] (see Fig. 4, *Background images in Recognition Memory*). Bayes factor provided moderate-to-strong evidence for the null hypothesis (*BF*_10_ = 0.103, ±0.18%), supporting the lack of an agency effect on recognition memory for background images.

#### Recognition Memory for Item Images

Recognition memory for item images also showed no significant difference between the active and yoked conditions. The mean score for the active condition (*M* = 0.570, *SD* = 0.258) was not significantly different from the yoked condition (*M* = 0.593, *SD* = 0.249), *t*(147) = 0.824, *p* = .411, 95% CI [−0.028, 0.069] (see Fig. 4, *Item images in Recognition Memory*). Bayes factor provided moderate evidence for the null hypothesis (*BF*_10_ = 0.128, ±0.15%), indicating no significant agency effect on recognition memory for item images.

#### Character-Source Memory for Background Images

Character-source memory for background images showed no significant difference between the active and yoked conditions. The mean score for the active condition (*M* = 0.460, *SD* = 0.353) was not significantly different from the yoked condition (*M* = 0.484, *SD* = 0.355), *t*(135) = −0.797, *p* = .427, 95% CI [−0.102, 0.044] (see Fig. 4, *Background images in Character Source Memory)*. Bayes factor provided moderate evidence for the null hypothesis (*BF*_10_ = 0.130, ±0.14%), suggesting no significant agency effect on character-source memory for background images.

#### Character-Source Memory for Item Images

Character-source memory for item images also showed no significant difference between the active and yoked conditions. The mean score for the active condition (*M* = 0.351, *SD* = 0.282) was not significantly different from the yoked condition (*M* = 0.405, *SD* = 0.248), *t*(135) = −0.174, *p* = .862, 95% CI [−0.061, 0.051] (see Fig. 4, *Item images in Character Source Memory*). Bayes factor provided strong evidence for the null hypothesis (*BF*_10_ = 0.0968, ±0.18%), indicating strong support for the lack of an agency effect on character-source memory for item images.

## General Discussion

This study investigated whether agency—the ability to make and act upon choices—enhances memory performance in young children aged 4 to 7 years. Across two experiments focusing on item recognition and binding, we found that children’s memory performance was similar regardless of whether they did or did not make choices. Our results align with previous research indicating that agency has little to no effect on memory at young ages. While studies involving older children and adults often—but not always—report memory enhancements associated with choice-making (e.g., Ding et al., 2021; Murty et al., 2015; Ruggeri et al., 2019), similar benefits are inconsistently observed in younger children (e.g., McComas et al., 1997). Also, in word-learning studies, toddlers sometimes show better learning in passive contexts (Ackermann et al., 2020; Bothe et al., 2024), whereas others report no difference between active and passive conditions (Sivridag & Mani, 2024). These findings underscore that agency effects may vary not only by age but also by the specific cognitive or linguistic domain being tested. However, it is important to note that the absence of an observed effect in our particular narrative-based paradigm does not preclude the possibility that agency might benefit memory in younger children under different task structures or feedback conditions. This pattern suggests that developmental factors play a significant role in the relationship between agency and memory. To contextualize our findings within the broader literature, Table 1 summarizes studies examining the effects of choice-making on memory across different age groups. The table highlights that the mnemonic benefits of the agency are more consistently observed in older children and adults.

Several cognitive and developmental factors could explain why age serves as a boundary condition for the effects of choice on memory. One factor is the development of cognitive control and executive functions. Cognitive control, including attention regulation, working memory, and inhibitory control, improves significantly during early childhood (Luna et al., 2015). Younger children might not have the executive functions necessary to utilize agency during learning tasks. The ability to plan, monitor, and adjust one’s behavior—an important aspect of benefiting from choice-making—may be underdeveloped in children aged 4 to 7 (Best & Miller, 2010; Karr et al., 2018; Munakata et al., 2012). The emergence of metacognitive abilities is another important consideration. Metacognition allows individuals to reflect on and regulate their cognitive processes. Older children and adults can use metacognitive strategies to enhance learning, such as recognizing the importance of paying attention to choices and their consequences; however, younger children may lack these metacognitive skills, limiting their ability to leverage agency for memory enhancement (Ghetti & Fandakova, 2020). Understanding of causal relationships also develops with age (Sobel et al., 2004; Sobel & Kirkham, 2006). Younger children do not fully grasp the causal relationship between their choices and subsequent events, reducing the impact of agency on memory encoding. In contrast, older children are more likely to understand that their decisions have meaningful consequences, which can enhance engagement and memory (Katzman & Hartley, 2020).

Recent research by Li et al. (2024) provides further insight into how agency influences memory; adults who exercised agency by making choices during an interactive narrative showed personalized memory patterns. The agency led to more significant individual variability in which events were remembered, suggesting that making choices can personalize memory by activating self-relevant information. This personalization effect indicates that agency may change how fundamental determinants of episodic memory operate during naturalistic experiences. However, the personalization of memory through agency observed in adults may not extend to younger children. Young children might not have developed self-referential processing or the ability to effectively integrate self-relevant information into their memories. The cognitive and metacognitive skills required to personalize memory through agency likely mature with age, which could explain why younger children in our study did not show enhanced memory performance when given choices.

As shown in Table 2, the benefits of choice-making on memory are not uniformly present across all ages or memory types. The findings of agency-based enhancement of memory in adults suggest that specific cognitive prerequisites—such as advanced executive functions, metacognition, and a deeper understanding of cause-and-effect relationships—are necessary for agency to positively impact memory.

Understanding that age is a boundary condition for the effects of choice on memory has practical implications. Educational strategies that incorporate agency and choice-making may need to be tailored to the developmental stage of learners. For younger children, alternative approaches that do not rely heavily on agency might be more effective in enhancing memory and learning. However, some research suggests that modifying how choices are presented could help younger children to benefit from the agency. Ruggeri et al. (2019) found that when children were given repeated opportunities to make choices or allowed to select multiple times in succession, even younger children showed improvements in certain types of memory tasks, such as visual recognition and remembering names. Therefore, by designing tasks that involve repeated decision-making or allowing children to make multiple choices consecutively, it may be possible to enhance the mnemonic benefits of agency in younger learners. As children mature and develop the necessary cognitive skills, incorporating more complex choice-making activities could become increasingly beneficial.

Several limitations of our study should be acknowledged. The nature of the choices and the feedback provided might not have been sufficient to engage younger children fully. Immediate and salient feedback is known to reinforce learning, and its absence might have diminished the potential benefits of agency (Hon & Yeo, 2021). Additionally, motor engagement, known to facilitate memory encoding, was limited since the actions were performed by parents rather than the children themselves. Studies have shown that active involvement and motor processes can enhance memory (Kinder & Buss, 2021; Yebra et al., 2019). The lack of direct motor engagement in our experimental design might have reduced potential benefits associated with active involvement.

One reason we limited explicit feedback statements (e.g., “You chose the yellow door, so now the living room appears”) was to preserve the immersive, child-friendly nature of the task. By inviting children to act on behalf of the character—rather than operating as external observers—we hoped to keep them engaged in the storyline without repeatedly “breaking the fourth wall.” Although this may have dampened the child’s awareness that their own choice caused the outcome, we felt it would encourage a more naturalistic, playful experience. Future studies could compare this approach with a version that provides clear, causal feedback after each choice (e.g., “Because you selected X, now Y occurs”) to determine whether more explicit feedback enhances the sense of agency and yields stronger memory benefits.

Another important consideration is that our task used longer, narrative-based segments rather than the brief post-choice stimuli commonly employed in prior laboratory studies. If the memory advantage of choice is relatively short-lived—because it helps focus attention immediately before stimulus presentation—a more extended storyline could overshadow or dilute that effect. Once children shifted their focus to subsequent parts of the cartoon, any short-term attentional benefit from having chosen may have faded. Future research could manipulate the length or timing of post-choice segments to clarify whether briefer episodes are necessary for observing a strong mnemonic benefit from choice, especially in younger children.

Moreover, while our sample size provided sufficient power to detect an overall moderate effect between the active and yoked conditions for children aged 4 to 7, it was not large enough to detect subtle or smaller effects within specific age subgroups (e.g., comparing 4-year-olds vs. 5-year-olds). In other words, once our overall sample is subdivided by each individual age, the effective sample size per group is substantially reduced, which diminishes our ability to detect more subtle age-related differences. Thus, it remains possible that small or age-specific effects of agency on memory may emerge more clearly at certain points between ages 4 and 7 but were not observed here due to in sufficient power at each individual age. Future work with larger, more balanced samples across this age range would be necessary to identify such fine-grained developmental effects. However, due to practical constraints (e.g., budget, time, recruitment limitations), we were unable to recruit additional participants in the current study.

Individual differences in cognitive abilities might also moderate the impact of agency on memory. Markant (2020) found that individuals with higher working memory capacity benefited more from choice-making. While we assessed cognitive abilities using standardized tests like the Kaufman Brief Intelligence Test (KBIT) in Experiment 2, our sample size was insufficient to analyze how these differences might have influenced the results. Future research with larger samples could explore how individual cognitive capacities interact with agency in young children’s memory performance.

Future research should explore developmental trajectories by conducting studies to examine how the effects of agency on memory evolve with age, identifying the developmental milestones associated with the emergence of these benefits. Modifying task designs to include more immediate feedback, meaningful choices, and motor involvement might help determine whether these factors enable younger children to experience memory benefits from agency. Investigating how cognitive capacities such as working memory and metacognition interact with agency could provide a more nuanced understanding of when and for whom choice-making enhances memory. Additionally, comparing young children’s performance with adult samples on tasks that share a similar interactive or narrative structure (Houser et al., 2022; Rotem-Turchinski et al., 2018) could clarify whether the selective benefits of agency—such as improved temporal order memory—are contingent on participants’ developmental level or on specific task features.

In conclusion, our study indicates that agency does not enhance memory performance in children aged 4 to 7 years, suggesting that age can be a boundary condition for the mnemonic benefits of choice-making. Developmental factors, including the maturation of cognitive control, metacognition, and understanding of causal relationships, likely contribute to this boundary effect. Recognizing when agency is most effective will help educators and researchers design interventions that support memory and learning across different age groups. Nevertheless, our findings are specific to the narrative-based context and task parameters used here. Future work should test whether variations in feedback, more immediate consequences of children’s choices, or different age subgroups might reveal benefits that were not detected in the present design.

## Supplementary Material

Peer Review Communication

Supplementary Materials

Peer Review Communication

Download: https://collabra.scholasticahq.com/article/137316-the-limits-of-agency-young-children-s-memory-may-not-benefit-from-choice/attachment/280967.docx?auth_token=vdMSHp730W1TDPgHEBLQ

## Figures and Tables

**Figure 3a.**
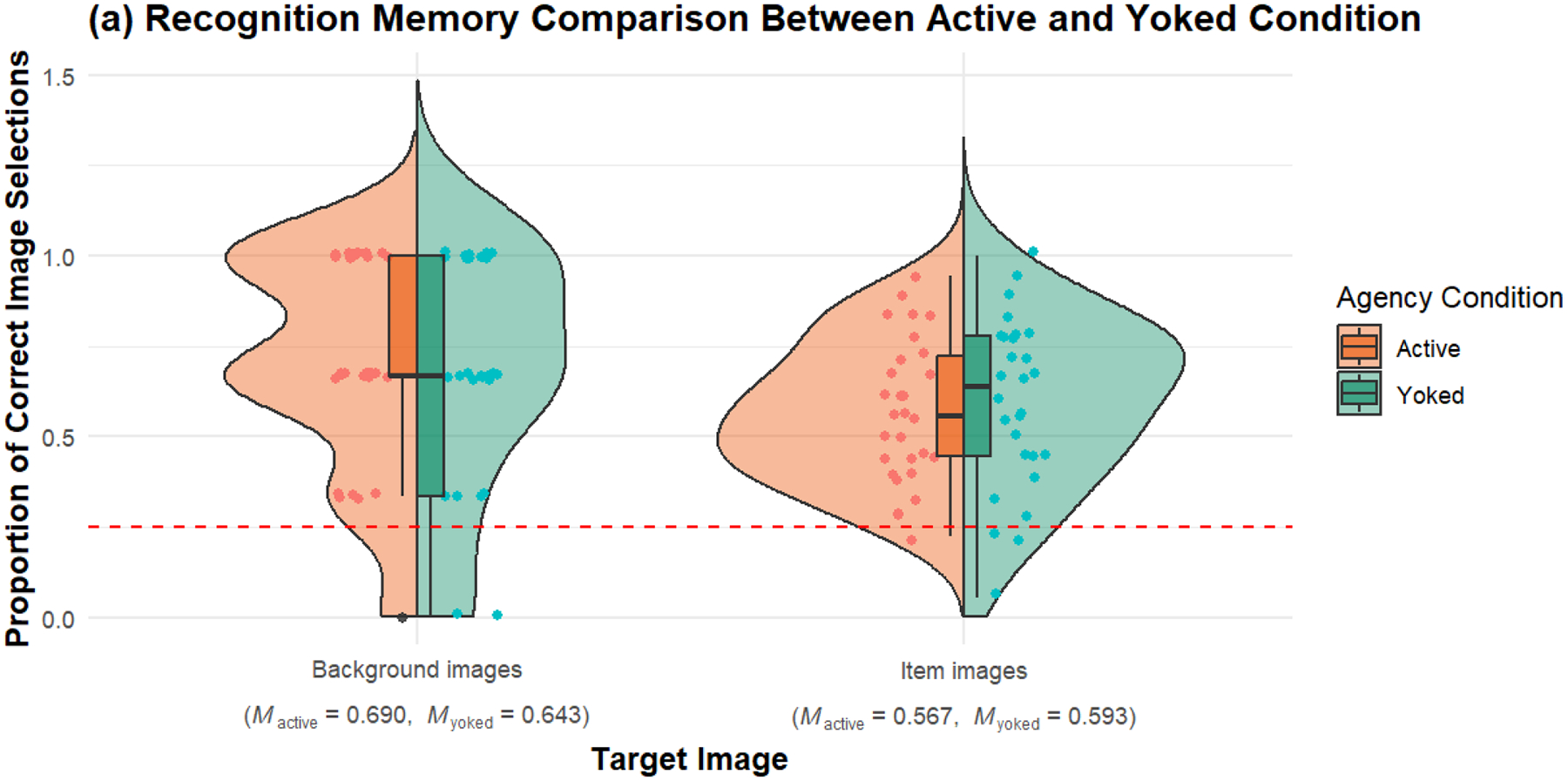
Comparison of recognition memory performance between active and yoked conditions in Experiment 1. The split-violin plots display the distribution of correct image selection proportions for both item and background images across the two conditions. The plot combines the data for item and background images, with the left side of the violin representing the active condition and the right side representing the yoked condition. The points within the violins indicate individual data points, and the overlaid boxplots represent the interquartile range and median of the differences. The red dashed line marks the chance level (0.25) for a 4AFC task where each of the four options has an equal chance of being correct.

**Figure 3b.**
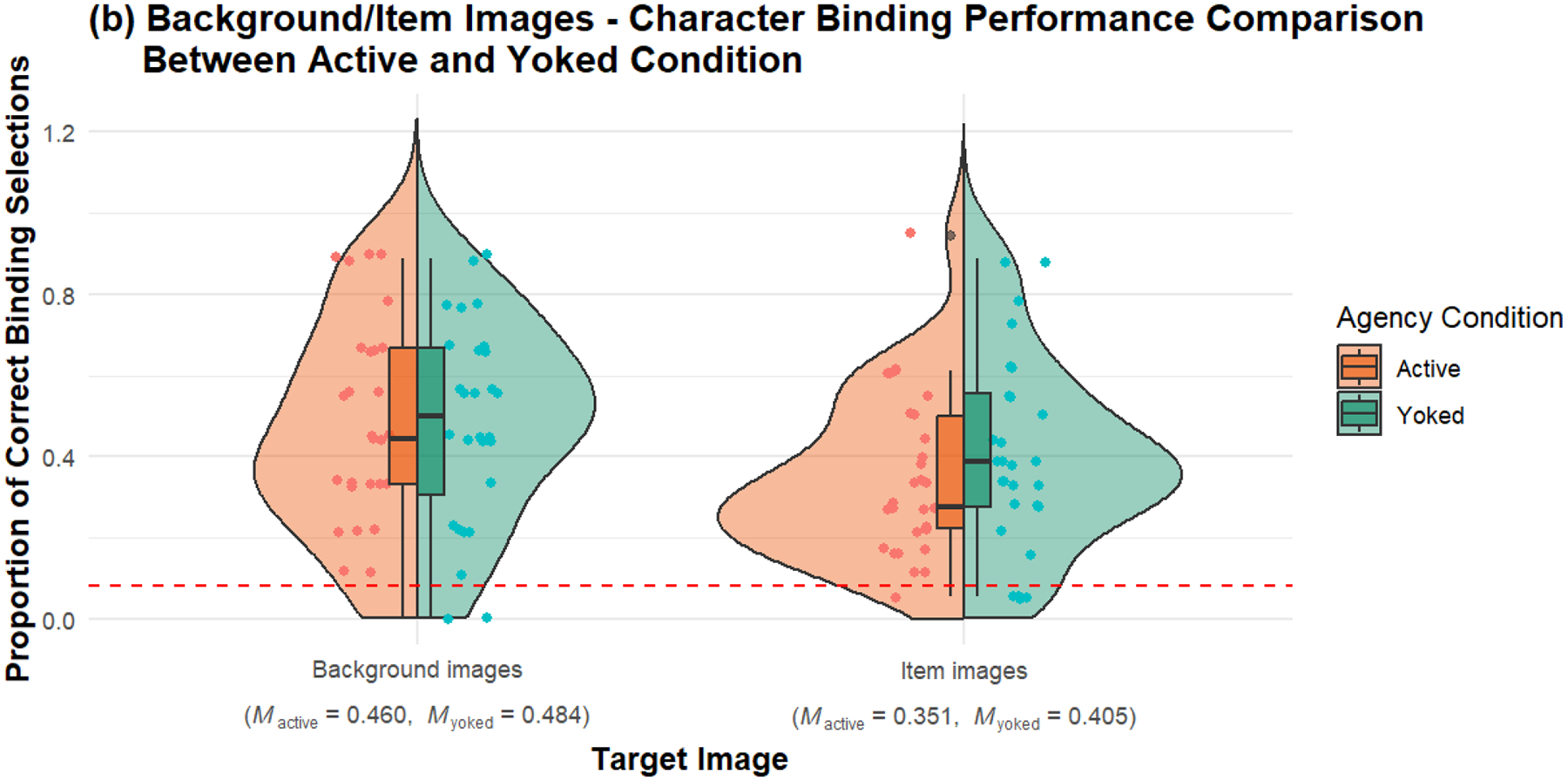
Comparison of background/item images and character binding performance between active and yoked conditions in Experiment 1. The split-violin plots display the distribution of correct binding selections for both background and item images across the two conditions. The left side of each violin represents the active condition, while the right side represents the yoked condition. The points within the violins indicate individual data points, and the overlaid boxplots represent the interquartile range and median of the differences. The red dashed line at 0.0825 indicates the chance level, which reflects the combined probability of correctly answering a recognition question (chance: 0.25) and a character source question (chance: 0.33), resulting in a total chance level of 0.0825.

**Table 1.** Summary of the studies testing choice-making activity helps memory. It highlights the varied effects of choice-making on memory across different age groups and memory types, emphasizing the conditions under which choice-making can enhance memory performance.

Study	N (Sample Size)	Age Range	Active Choice Condition	Yoked/Passive Condition	Experimental Design	Delay	Type of Memory Tested	Effect of Choice on Memory
Ding et al. (2021)	84 total (42 in Exp.1, 42 in Exp.2)	Young adults	Freely chose 1 of 2 “?” occluders to reveal an image.	Occluder indicated by an arrow (no autonomy).	Within-subjects	24 hours	Exp.1: Intentional Exp.2: Incidental	**Yes**: Choice enhanced 24-hour recognition in both
Hon & Yeo (2021)	111 total (60 in Exp.1, 51 in Exp.2)	Young adults	Short delay (100ms) + movement congruence → strong sense of agency.	Longer delay (900ms) or incongruence → weaker agency.	Within-subjects	Immediate	Incidental recognition	**Mixed**: Benefit only in strong-agency trials
Houser et al. (2022)	274 total (114 in Exp.1, 160 in Exp.2)	Ages 18–45 (adults)	Freely explored items in a text-based interactive “search for a key” game (agentive group).	Followed the identical sequence of item-clicks from a matched agentive participant (no personal choice).	Between-subjects	Exp.1: 0 & 24 hrs Exp.2: 0 hrs	Temporal order memory, item–spatial context, item-description memory	**Mixed**: Enhanced temporal order memory only, with no effect on item-description or spatial memory
Katzman & Hartley (2020)	96 total (31 children, 31 adolescents, 34 adults)	Ages 8–25	“Captain” trials: chose planet (high vs. low reward).	“Autopilot”: planet pre-selected (no agency).	Within-subjects	24 hours	Recognition and source memory	**Mixed**: Helped memory only when choice had high utility
Markant (2020)	200 total (100 in Exp.1, 100 in Exp.2)	Young adults	Chose which face to reveal (hierarchy task).	Observed pseudo-random or prior active sequences (no personal control).	Within-subjects	Exp.1: 0 & 6–8 days Exp.2: 0 days	Transitive inference	**Mixed**: Benefit only for high working-memory participants
McComas et al. (1997)	84 total (52 in Exp.1, 32 in Exp.2)	~6–7 yrs (Exp.1), ~6–9 yrs (Exp.2)	“Active choice–active movement” or “active choice–passive movement.”	“Passive choice–active movement” or “passive choice–passive movement.”	Between-subjects (2×2)	Immediate	Spatial memory of visited locations	**No**: Movement helped, but mere choice did not
Murty et al. (2015)	22 adults (fMRI), 16 adults (replication)	Young adults	Freely chose which occluder to remove (trial-unique object beneath).	Computer indicated which occluder to remove (no agency).	Within-subjects	24 hours	Recognition (old/new)	**Yes**: Better memory for chosen items
Rotem-Turchinski et al. (2018)	58 total (46 final)	Young adults	Interactive movie clips: participant believes they choose the storyline’s continuation.	Computer “chose” the clip’s outcome (participant just continued).	Mixed (within + between)	2 days or 1 week	Incidental recognition (details, choices)	**Yes**: Active choice improved detail memory
Ruggeri et al. (2019)	171 total (3 experiments)	Ages 5–11	Decided order/duration of studying objects; controlled own viewing (active).	“Replay” of another’s active sequence (no personal control).	Within-subjects	~1 week (Exp.1 & 2), Immediate (Exp.3)	Object/spatial/labels recognition	**Mixed**: Stronger benefit in children ≥6 yrs
Tsuji & Imaizumi (2022)	56 total (28 in Exp.1, 28 in Exp.2)	Young adults	Voluntary action (key press) → stimulus onset (manipulated sense of agency).	Baseline/no-action: stimulus appeared automatically.	Within-subjects	Immediate	Recognition (recollection/familiarity)	**No**: No agency effect on recognition
Current study	94 total (57 in Exp.1, 37 in Exp.2)	Ages 4–7	Child chose which of two cartoon “endings” to watch (agency).	“Yoked”: ending was automatically selected (no choice).	Exp.1: Between Exp.2: Within	6–8 days	Recognition (item/background) + Binding (char.–source)	**No**: No memory benefit from choice

**Table 2.** Demographics of All Participants

	Total (n = 57)		Total (n = 37)	
	Experiment 1		Experiment 2	
	Count	Percent	Count	Percent
Race				
*Asian or Asian American*	4	7.02%	4	10.81%
*Black or African American*	10	17.54%	5	13.51%
*Mixed*	6	10.53%	3	8.11%
*White or Caucasian*	37	64.91%	25	67.57%
Ethnicity				
*Not Latinx*	51	89.47%	34	91.89%
*Latinx*	6	10.53%	3	8.11%
Gender				
*Female*	36	63.16%	20	54.05%
*Male*	21	36.84%	17	45.95%
Age				
*Four years (4.00–4.99)*	14	24.56%	8	21.62%
*Five years (5.00–5.99)*	16	28.07%	10	27.03%
*Six years (6.00–6.99)*	18	31.58%	9	24.32%
*Seven years (7.00–7.99)*	9	15.79%	10	27.03%
Income				
*Less than $15,000*	0	0	1	2.70%
*$15,000 to $34,999*	2	3.51%	2	5.41%
*$35,000 to $49,999*	4	7.02%	0	0.00%
*$50,000 to $74,999*	12	21.05%	6	16.22%
*$75,000 to $99,999*	4	7.02%	4	10.81%
*$100,000 to $124,999*	11	19.30%	3	8.11%
*$125,000 to $149,999*	5	8.77%	5	13.51%
*$150,000 or more*	19	33.33%	16	43.24%

## Data Availability

All the Qualtrics stimuli, participant data, and analysis scripts can be found on this paper’s project page at https://osf.io/fyhd8/.
