# Supplementary material for: The Limits of Agency: Young Children’s Memory May Not Benefit From Choice
Source: Collabra Psychol. Author manuscript; Available in PMC 2025 Jul 5. (PMC12228495; doi:10.1525/collabra.137316)
Supplement: Peer Review Communication [file NIHMS2093002-supplement-Peer_Review_Communication.docx]

**Peer Review and Communication History**

**MS Title**: The Limits of Agency: Young Children’s Memory May Not Benefit from Choice

**Author Names**: Naoya Tani, Nora S. Newcombe & Ingrid R. Olson

**Submitted:** Oct 20, 2024

**Editor First Decision**: Revise & Resubmit

Dec 9, 2024

Dear Dr. Naoya Tani,

I have now received two very thorough reviews of your manuscript, “The Limits of Agency: Young Children’s Memory May Not Benefit from Choice”, from experts in the field. I also independently read the manuscript before consulting these reviews. The reviewers had mostly positive reactions to your manuscript. I agree that your manuscript has important strengths and also that there are some issues that need to be addressed. I therefore encourage you to submit a revised version for further consideration at Collabra: Psychology.

In my view, the chief issue is that the sample sizes are small, so I would ask that you be very transparent in your revision about what you can and cannot say with certainty given the small sample.

In your resubmission, please include a document with a point-by-point response to both the points I list here and the reviewers’ comments, outlining each change made in your manuscript or providing a suitable rebuttal. Please ensure that your revised files adhere to our author guidelines, and that the files are fully copyedited/proofed prior to upload. Please also ensure that all necessary copyright permissions have been obtained. This may be the last opportunity for major editing, therefore please fully check your file prior to re-submission.

If you have any questions or difficulties during this process, please contact the editorial office at [editorialoffice@collabra.org](mailto:editorialoffice@collabra.org).

We hope you can submit your revision within the next six weeks. If you cannot make this deadline, please let us know as early as possible.

Sincerely,
Brenna Hassinger-Das

# Reviewer 1

##### Rating scale questions

|  | Strongly Disagree | Disagree | Neutral | Agree | Strongly Agree |
| --- | --- | --- | --- | --- | --- |
| The study/studies in this manuscript have strong construct validity (good measures and/or manipulations of the constructs the authors wish to study). (Choose “Neutral” if this is not an empirical manuscript) |  |  | ✔ |  |  |
| The study/studies in this manuscript have strong statistical validity (appropriate statistical tests, assumptions are clear and reasonable, no statistical errors, appropriate statistical inferences, etc.). (Choose “Neutral” if this is not an empirical manuscript) |  | ✔ |  |  |  |
| The study/studies in this manuscript have strong internal validity (any causal claims or implications are well-justified, alternative explanations are thoroughly considered, etc.). (Choose “Neutral” if this is not an empirical manuscript, or no causal claims are made or even vaguely implied.) |  |  |  | ✔ |  |
| The study/studies in this manuscript have strong external validity (authors appropriately constrain their conclusions based on the limits of the generalizability of their findings to other contexts (including from lab to real world), other populations, other stimuli or measures, etc.) |  |  |  | ✔ |  |

##### Open response questions

### Please write your review here. The author(s) will see this review. Your identity will not be revealed to the authors unless you also include your name (i.e., sign your review) in this box. It is up to you whether to reveal your identity or not, either is fine.

The manuscript presents two experiments that compared 4–7-year-olds’ memory for events between two conditions: an active and a yoked condition. In both experiments, there was no benefit of active over yoked, which is partly in contrast with previous experiments.

I enjoyed reading the manuscript (the writing is great!), but some aspects of the work remained unclear to me, and others (particularly the low sample sizes) lowered my enthusiasm. Perhaps these points can be addressed in a revision, which may need additional data.

Major Points:

1. I would like to see a much clearer description of what the commonalities and differences are between the current experiment and previous experiments using an active vs yoked manipulation. This description should include the conditions, instructions, stimuli, and memory test measures. Perhaps a table akin to the current Table 2 could be the way to go.
   a. The description of the yoked condition remained particularly unclear to me. What was the cover story for the kids in the yoked condition? what exactly did they see? In active vs yoked designs, the yoked condition is the hard one to design properly, hence the readers would need way more information to evaluate it.
   b. It seems like the choices that kids in the active condition can make are random (i.e., not semantically or otherwise meaningful). They also do not seem to change anything in the way the story progresses. Is that true or a misunderstanding on my part? If it is true, it would be an explanation for the lack of an effect.
   c. Recognition memory test: I did not understand the difference between foil 1 and foil 2 (e.g., p. 8): “Each recognition memory question included four options: correct (the image seen in the post-cartoon story from session 1), lure (like the correct image but not seen in session 1), foil (not seen in session 1), and foil 2 (similar to foil 1 but not seen in session 1).”??
2. The authors do not state hypotheses and, consequently, there is no justification of statistical power to test them. Why is that? Given that the sample sizes are low, I wonder how reliable the results are.
3. The authors speculate about insufficient cognitive/metacognitive abilities in children as the most likely reason for the lack of an effect. I agree, but such an interpretation could be made much more forcefully with a bigger sample that would enable analyses regarding age-related differences. The current age range (4–7) is big enough to expect meaningful developmental. Is there any chance the authors could test more children and perform these analyses?

Minor Points:

1. I could not find: “Li, X., Ni, N. K., Born, S., Gualano, R., Lee, I., Bellana, B., & Chen, J. (2024). Agency personalizes episodic memories. Preprints.”

# Reviewer 2

##### Rating scale questions

|  | Strongly Disagree | Disagree | Neutral | Agree | Strongly Agree |
| --- | --- | --- | --- | --- | --- |
| The study/studies in this manuscript have strong construct validity (good measures and/or manipulations of the constructs the authors wish to study). (Choose “Neutral” if this is not an empirical manuscript) |  |  |  | ✔ |  |
| The study/studies in this manuscript have strong statistical validity (appropriate statistical tests, assumptions are clear and reasonable, no statistical errors, appropriate statistical inferences, etc.). (Choose “Neutral” if this is not an empirical manuscript) |  |  |  |  | ✔ |
| The study/studies in this manuscript have strong internal validity (any causal claims or implications are well-justified, alternative explanations are thoroughly considered, etc.). (Choose “Neutral” if this is not an empirical manuscript, or no causal claims are made or even vaguely implied.) |  |  |  | ✔ |  |
| The study/studies in this manuscript have strong external validity (authors appropriately constrain their conclusions based on the limits of the generalizability of their findings to other contexts (including from lab to real world), other populations, other stimuli or measures, etc.) |  | ✔ |  |  |  |

##### Open response questions

### Please write your review here. The author(s) will see this review. Your identity will not be revealed to the authors unless you also include your name (i.e., sign your review) in this box. It is up to you whether to reveal your identity or not, either is fine.

Summary: This study examines the effect of active control on memory among 4-7-year-old children. The authors created a naturalistic task to examine the effect of active choice-making in the context of narratives (cartoon stories). In the Active condition participants made a single choice which determined the direction of the narrative, while in the Yoked condition participants observed a choice and the ensuing narrative. Two online experiments were conducted. Experiment 1 (N=57) involved a between-subjects manipulation of choice condition while Experiment 2 (N=37) involved a within-subjects design. Outcomes of interest included item recognition, background image recognition, character source memory for background images, and character source memory for items. No significant effects of active control were found in either experiment, with additional Bayesian analyses indicating varying degrees of support for the null hypothesis of no difference between conditions. The authors suggest that mnemonic benefits of choice may not be present among younger children, possibly due to other factors such as the maturation of cognitive control, metacognition, or causal reasoning.

The paper is generally well-organized and written in a clear manner. The study design and analysis methods appear to be appropriate for the research question and to be sufficiently well-documented. Examining the effects of choice in the context of narrative stories is in some ways a much more naturalistic setting than some of the relevant earlier studies and a nice direction to take this line of work.

There are however some key limitations of this work:

1. The task used here–while more naturalistic–is also different in many ways from previous studies examining the effect of active choice on memory, including in children. A major difference is that in many of the previous studies, choices are followed by a relatively brief stimulus presentation. If the benefit of active choice is short-lived (e.g., because it allows participants to better align stimulus presentation with their attentional resources), then you might expect that effect to dissipate with longer episodes that unfold after the choice where there is more opportunity to encode the stimuli.

The authors acknowledge some of the differences between their task and previous work in the discussion, but still seem to draw an overly broad conclusion that “agency does not enhance memory performance in children aged 4 to 7 years”–it’s not clear the authors are finding a developmental “boundary” of the effect observed in most previous studies. The present work instead seems to address a much more open question of whether minimal active control (a single consequential choice) leads to an advantage in these narrative contexts. Given that, it would be useful to focus more of the background and discussion on studies with a similar narrative or sequential structure (e.g., Rotem-Turchinski et al., 2019; see also Houser, Tompary, & Murty, 2022) to establish a clearer expectation for whether a benefit is seen from agency among adults in comparable tasks.

Houser, T. M., Tompary, A., & Murty, V. P. (2022). Agency enhances temporal order memory in an interactive exploration game. Psychonomic Bulletin & Review, 29(6), 2219–2228. <https://doi.org/10.3758/s13423-022-02152-7>

2. Given the many differences between the present study and the studies listed in Table 2, the objectives of the present study did not come through clearly to me. Previous studies have focused on children in a similar age range, and the present sample isn’t large enough to detect developmental differences within the span of 4-7 years (which previous work suggests is a time when the effect of agency might emerge). Conceptually the idea of an age-based “boundary condition” needs more justification, since the effect likely depends on other developmental factors and the task characteristics. The authors point to some reasonable factors which might be related to the effect (e.g., metacognition, working memory, causal reasoning), but the present study doesn’t appear to be designed to provide insight into those relationships (it’s not clear why some measures of intellectual ability were collected but not analyzed).

# Minor points

- pg. 3: “This effect likely occurs because making choices increases cognitive engagement during the learning process” – By cognitive engagement, do you mean attention? Would be helpful to be more precise.
- Pg. 4: “However, despite the importance of understanding how agency affects learning in early childhood, there is a paucity of research on children under the age of 8.” – I think this is mostly fair, but might consider these recent studies that have looked at younger age range:

Ackermann, L., Lo, C. H., Mani, N., & Mayor, J. (2020). Word learning from a tablet app: Toddlers perform better in a passive context. PLOS ONE, 15(12), e0240519. <https://doi.org/10.1371/journal.pone.0240519>

Bothe, R., Isbaner, S., Chen, X., Kagan, I., Gail, A., & Mani, N. (2024). Little scientists & social apprentices: Active word learning in dynamic social contexts using a transparent dyadic interaction platform.

Sivridag, F., & Mani, N. (2024). Children’s Word Learning from Socially Contingent Robots Under Active vs. Passive Learning Conditions. Proceedings of the 2024 ACM/IEEE International Conference on Human-Robot Interaction, 669–677. <https://doi.org/10.1145/3610977.3634931>

- Pg. 16: I was unsure why Table 2 appears in the general discussion, when many of the studies are also presented as background in the Introduction.

**Author Response**
Jan 21, 2025

Dear Dr. Hassinger-Das,

We thank you and the reviewers for your thoughtful and constructive feedback on our manuscript, “The Limits of Agency: Young Children’s Memory May Not Benefit from Choice.” We greatly appreciate the time and effort invested in reviewing our submission. We have carefully revised the manuscript in response to each comment you and the reviewers raised, and we believe the changes have strengthened our work.

In what follows, we provide a detailed, point-by-point response. We have quoted or summarized each reviewer’s comment to facilitate clarity, followed by our response. We have also indicated where the corresponding revisions have been made in the revised manuscript.

We hope that our revised manuscript and this response letter address all concerns. We look forward to your further feedback and the possibility of publishing our work.

1. Reviewer 1 Comments
   1.1. Major comments
        1.1.1. Clarification of active vs. yoked manipulation differences

               **Reviewer 1:**
               “I would like to see a much clearer description of what the commonalities and differences are between the current experiment and previous experiments using an active vs yoked manipulation. Perhaps a table akin to the current Table 2 could be the way to go.”

               **Response:**
               We appreciate this suggestion and have created a new table (see Table 2) that more clearly illustrates how our experimental design compares to previous studies using active versus yoked manipulations. In this revised table, we highlight each study’s sample size (N), age range, nature of the “active” and “yoked” conditions, experimental design (between- vs. within-subjects), delay, type of memory assessed, and whether or not choice benefited memory. We believe this format will help readers quickly identify the core similarities and differences among prior work and situate our findings accordingly.
        1.1.2. Description of the yoked condition

               **Reviewer 1:**
               What was the cover story for the kids in the yoked condition? What exactly did they see? In active vs yoked designs, the yoked condition is the hard one to design properly, hence the readers would need way more information to evaluate it.

               **Response:**
               We appreciate your concern about the clarity of the yoked condition, particularly regarding what the children saw and experienced. In the revised manuscript, we have added a detailed explanation of how the yoked condition was introduced and implemented.
               Children in the yoked condition had a clear “cover story”: the cartoon character had already decided which path to follow, leaving no perceived opportunity for the child to choose. We reinforced this idea during the brief practice session (using a different cartoon clip) so that participants knew to expect automated decisions throughout the main study. This approach helped ensure that children did not feel deprived of an opportunity to choose but rather understood that in this particular game, the character was simply making its own decisions.

1.1.3. Nature of choices in the active condition

       **Reviewer 1**:
       “It seems like the choices that kids in the active condition can make are random (not semantically meaningful). They do not seem to change anything in the way the story progresses. Is that true?”

       **Response:**
       We appreciate the reviewer’s concern. In our study, each choice in the active condition *did* lead to a *different* follow-up scene with its own unique background and items. For instance, if children chose the ‘outside’ option, they might see a backyard scene with a red ball and a green bench, whereas choosing the ‘inside’ option would show a living-room scene with different items (e.g., a purple teddy bear and a red toy car). This ensured that the child’s decisions influenced what was encoded into memory during the learning phase. We have clarified this aspect in the Methods section (see page 9-11).

       It is true that children could, in principle, make random selections. However, our exclusion criteria stipulated that if a child disengaged from the task (e.g., consistently choosing the same option without paying attention or otherwise showing signs of non-compliance), we would not include those data in our final analysis. No participants met these criteria during data collection, suggesting that children were indeed engaged in making meaningful choices.

       Additionally, we prepared nine independent stories (three per cartoon). Each story had exactly one choice screen followed by a single “post-cartoon” segment—so one branch (e.g., “outside”) led to one unique set of background and items, while the other branch (e.g., “inside”) led to a different set. Thus, each choice directly determined what scene the children would see next, meaning their selections did influence the story events that were presented to them. We have clarified this aspect in the Methods section (see page 9-11).
1.1.4. Recognition memory test (foil 1 vs. foil 2)

       **Reviewer 1:**
       Recognition memory test: I did not understand the difference between foil 1 and foil 2 (e.g., p. 8): “Each recognition memory question included four options: correct (the image seen in the post-cartoon story from session 1), lure (like the correct image but not seen in session 1), foil (not seen in session 1), and foil 2 (similar to foil 1 but not seen in session 1).”??

       **Response:**
       Thank you for pointing out the need for clarification regarding the distinction between Foil 1 and Foil 2 in our recognition memory task. In our design, **both Foil 1 and Foil 2 are novel images that did not appear during encoding**. However, we included two different foils to ensure that children could not rely on partial memory or mere process of elimination.We have revised the manuscript to clarify this point (page 9-11), adding a concrete example to illustrate the difference between lure, Foil 1, and Foil 2.
1.1.5. Sample size, statistical power, and reliability of results

       **Reviewer 1:**
       The authors do not state hypotheses and, consequently, there is no justification of statistical power to test them. Given the sample sizes are low, I wonder how reliable the results are.

       **Response:**
       Thank you for highlighting these important considerations. We have clarified our central hypotheses, added a brief discussion of our power considerations, and expanded on our use of Bayesian analyses to address questions of effect size and evidence for the null. Specifically:

       **Explicit Statement of Hypotheses**
       In both Experiments 1 and 2, our primary hypothesis was that children who actively made choices (active/agency condition) would exhibit better memory performance than children who did not make their own choices (yoked condition). This hypothesis was grounded in prior findings that active choice can enhance declarative memory in older children and adults. We have now added an explicit statement of these hypotheses in the Introduction (page 7-8).

       **Justification for Sample Size and Power**
       *Bayesian Analysis and JZS Priors.* We employed the default Jeffreys–Zellner–Siow (JZS) prior within the BayesFactor package in R. The JZS prior is considered relatively noninformative and balanced for common t-tests, providing a fair test for moderate-to-large effect sizes. Had our sample size been too small to detect a meaningful effect, or had the prior been overly restrictive, we would likely have obtained Bayes factors close to 1.0 (i.e., inconclusive results). Instead, our Bayes factors consistently indicated moderate-to-strong evidence in favor of the null hypothesis. Thus, the data are more consistent with no effect—or only a very small effect—of agency on memory performance in this age group, rather than an artifact of low power. We have revised the Methods section in order to clarify our data analysis approach (page 13).
       *Limitations and Smaller Effects.* We acknowledge that our sample sizes may not reliably detect very small effects, and we do not exclude the possibility that a small benefit of agency could still exist. However, the current study design would likely have detected moderate-to-large effects similar to those observed in older children and adults. That we found consistent evidence for the null across two experiments underscores the reliability of our conclusion.
1.1.6. **Potential developmental differences and future data collection

       Reviewer 1:**
       Is there any chance the authors could test more children and perform age-related analyses? **Response:**
       We appreciate your suggestion to recruit additional participants for more fine-grained, age-related analyses. We agree that a larger sample size across the 4- to 7-year-old range would allow us to better explore developmental trajectories in the effects of agency on memory. Unfortunately, due to budget, time and recruitment limitations, we could not extend our data collection in this project. Nevertheless, we have revised our Discussion (page 23) to emphasize the need for future research with larger samples and expanded age ranges to better capture potential age-related changes. This will be an important next step for clarifying how the maturation of cognitive skills contributes to the relationship between agency and memory in early childhood.
1.2. Minor Comments
     1.2.1. **Reviewer 1**:
            I could not find: ‘Li, X., Ni, N. K., Born, S., Gualano, R., Lee, I., Bellana, B., & Chen, J. (2024). Agency personalizes episodic memories. Preprints.’

            **Response:**
            We have verified the citation and updated the reference list to include the correct link/DOI for the preprint (page 27).

2. Reviewer 2 Comments
   2.1. Major Comments
        2.1.1. **Task differences compared to previous studies**

               **Reviewer 2**:
               The task used here–while more naturalistic–is also different in many ways from previous studies examining the effect of active choice on memory, including in children. A major difference is that in many of the previous studies, choices are followed by a relatively brief stimulus presentation. If the benefit of active choice is short-lived (e.g., because it allows participants to better align stimulus presentation with their attentional resources), then you might expect that effect to dissipate with longer episodes that unfold after the choice where there is more opportunity to encode the stimuli.

               **Response:**
               Thank you for highlighting these important methodological differences. In our revised Discussion (pages 23), we have expanded our explanation of how our naturalistic, narrative-based design may diverge from more tightly controlled laboratory paradigms, particularly those that feature very brief post-choice stimulus exposures. We acknowledge that if the mnemonic benefit of active choice is relatively short-lived—potentially because it helps learners better focus their attentional resources on the upcoming stimulus—the advantage might indeed be diluted or overshadowed by a longer, unfolding narrative. This reduced impact could stem from children shifting their attention to the broader storyline rather than remaining focused on the immediate outcome of their choices.
        2.1.2. Scope of conclusions

               **Reviewer 2:**
               The authors still seem to draw an overly broad conclusion that ‘agency does not enhance memory performance in children aged 4 to 7 years.’ It’s not clear the authors are finding a developmental boundary of the effect observed in previous studies.

               **Response:**
               We have revised our conclusion statements throughout the Abstract and Discussion (pages 2 and 24) to be more conservative. Rather than broadly stating that “agency does not enhance memory,” we emphasize that in this particular context/task and with our current sample size, we did not observe a clear advantage for active choice. We also clarify that this finding does not necessarily rule out the possibility of agency effects in other contexts, tasks, or age groups.
        2.1.3. Clarifying objectives and developmental boundary conditions

               **Reviewer 2:**
               Given the many differences between the present study and the studies listed in Table 2, the objectives of the present study did not come through clearly to me. Previous studies have focused on children in a similar age range, and the present sample isn’t large enough to detect developmental differences within the span of 4-7 years (which previous work suggests is a time when the effect of agency might emerge). Conceptually the idea of an age-based “boundary condition” needs more justification, since the effect likely depends on other developmental factors and the task characteristics. The authors point to some reasonable factors which might be related to the effect (e.g., metacognition, working memory, causal reasoning), but the present study doesn’t appear to be designed to provide insight into those relationships (it’s not clear why some measures of intellectual ability were collected but not analyzed).

               **Response:**
               Thank you for highlighting these important points. We appreciate the opportunity to clarify our study’s objectives and to further justify our focus on children aged 4–7 years as a potential “boundary condition.” We address each concern below:

               **Study Objectives and Rationale**
               Our primary goal was to determine whether the effect of agency (i.e., choice-making) on memory—well-documented in adults and somewhat more variable in older children—generalizes to younger children under naturalistic, narrative-based conditions. Although previous work also includes children in a similar age range, much of it employs task structures (e.g., short, discrete trials, or reward-based designs) that differ from our more narrative-oriented approach. We chose this format to increase ecological validity and to explore how choice might manifest in a context that felt engaging for younger children. We have revised the manuscript to articulate more clearly that we sought to (a) replicate or refute the agency effect in a younger age group (4–7 years) and (b) explore whether a narrative-based approach would magnify or diminish any potential memory benefits from agency.

               **Detecting Developmental Differences Within 4–7 Years**
               We agree that pinpointing sub-group differences between four-, five-, six-, and seven-year-olds would be informative; however, we did not power our study to detect fine-grained age-related effects within this narrow range. Instead, we aimed for a sample size sufficient to detect a moderate-sized overall benefit of agency (vs. yoked) in young children. Our findings suggest that, at least in this combined 4–7-year-old age span and with our task design, the agency benefit is not present or is notably small. We have added a note in the Discussion now acknowledging that subtle age effects could exist but would require a more targeted, larger-sample approach to detect smaller, within-year differences.

               **Measures of Intellectual Ability and Lack of Analysis**
               Regarding the cognitive ability measures (e.g., KBIT-2, Woodcock–Johnson) that we collected, our intention was primarily to ensure children met a general developmental norm for inclusion in the study. We agree that investigating how working memory or general intelligence moderates the agency effect could be valuable. However, given our sample size and the study’s scope, we were not adequately powered to test higher-order interactions (e.g., Condition × Age × IQ) reliably. In the revised manuscript, we have added an explicit statement acknowledging this limitation and clarifying why these data were not analyzed in detail. We also propose that future studies, with larger samples and more comprehensive cognitive assessments, could elucidate how individual differences in these abilities might modulate the impact of agency on memory in younger learners.

               **Task Design and Future Directions**
               Lastly, we have expanded our Discussion to emphasize how specific task characteristics (e.g., length of the narrative, immediate vs. delayed feedback, degree of motor engagement) may be crucial moderators that we did not fully explore. We now articulate clearer suggestions for how future studies could manipulate these factors and/or measure children’s causal and metacognitive understanding to better delineate the conditions under which young children might benefit from agency.
        2.1.4. Additional references and relation to broader literature

               **Reviewer 2:**
               It would be useful to focus more of the background and discussion on studies with a similar narrative or sequential structure (e.g., Rotem-Turchinski et al., 2019; see also Houser et al., 2022).

               **Response**:
               We have incorporated additional references, including Houser et al. (2022), into the Discussion (page 24) and Table 2. We now compare our findings to these studies that employ narrative or sequential structures to further contextualize our results.
   2.2. Minor Comments
        2.2.1. **Reviewer 2:**
               pg. 3: “This effect likely occurs because making choices increases cognitive engagement during the learning process” – By cognitive engagement, do you mean attention? Would be helpful to be more precise.

               **Response:**
               We appreciate you pointing out the need to clarify what we mean by “cognitive engagement.” In the revised manuscript (page 3), we have replaced the phrase “increases cognitive engagement” with a more precise description, stating that making choices can heighten participants’ attention and more elaborative encoding thereby improving memory performance. We believe this change makes the underlying mechanism clearer and addresses the reviewer’s concern.
        2.2.2. **Reviewer 2:**
               Pg. 4: “However, despite the importance of understanding how agency affects learning in early childhood, there is a paucity of research on children under the age of 8.” – I think this is mostly fair, but might consider these recent studies that have looked at younger age range:

               Ackermann, L., Lo, C. H., Mani, N., & Mayor, J. (2020). Word learning from a tablet app: Toddlers perform better in a passive context. PLOS ONE, 15(12), e0240519. <https://doi.org/10.1371/journal.pone.0240519>

               Bothe, R., Isbaner, S., Chen, X., Kagan, I., Gail, A., & Mani, N. (2024). Little scientists & social apprentices: Active word learning in dynamic social contexts using a transparent dyadic interaction platform.

               Sivridag, F., & Mani, N. (2024). Children’s Word Learning from Socially Contingent Robots Under Active vs. Passive Learning Conditions. Proceedings of the 2024 ACM/IEEE International Conference on Human-Robot Interaction, 669–677. <https://doi.org/10.1145/3610977.3634931>

               **Response:**
               Thank you for pointing out these relevant studies on word learning in younger children. We have added a short statement to the Introduction (page 7) and a brief note in the Discussion (page 20) acknowledging that some investigations in toddlers and preschoolers reveal inconsistent outcomes regarding active versus passive learning. While these studies focus on language acquisition rather than memory binding or recognition, their results further suggest that agency does not uniformly enhance learning at younger ages, reinforcing our claim that the benefits of choice may be task- and domain-specific.
        2.2.3. **Reviewer 2:**
               Pg. 16: I was unsure why Table 2 appears in the general discussion, when many of the studies are also presented as background in the Introduction.

               Response:
               Thank you for pointing out your uncertainty about the placement of Table 2. We have now moved Table 2 to the Introduction section, where many of the referenced studies are first mentioned. This change aligns the table more closely with the background literature and should help readers see how the prior findings inform our research questions. We agree that presenting the summarized studies earlier in the manuscript clarifies the rationale for our focus on younger children and how our experiments build on existing work.

Once again, we thank both reviewers and you for thoroughly evaluating our work. We believe that the revisions made in response to these points have improved the manuscript’s clarity and the interpretation of our findings. We appreciate your consideration of our revised submission and look forward to your further feedback.

**Editor Second Decision**: Revise & Resubmit

Mar 5, 2025

Dear Dr. Naoya Tani,

I have now received two very thorough reviews of your manuscript, “The Limits of Agency: Young Children’s Memory May Not Benefit from Choice”, from experts in the field. I also independently read the manuscript before consulting these reviews. The reviewers had mostly positive reactions to your manuscript. I agree that your manuscript has important strengths and also that there are some issues that need to be addressed. I therefore encourage you to submit a revised version for further consideration at Collabra: Psychology.

In your resubmission, please include a document with a point-by-point response to both the points I list here and the reviewers’ comments, outlining each change made in your manuscript or providing a suitable rebuttal.

As stated by Reviewer 1, I ask the authors to address the issue of small sample sizes more clearly in the Limitations.

I also agree with Reviewer 1 that I’m still not 100% convinced that the choices the children were able to make in the active condition were (perceived to be) meaningful. I ask the authors to explain in the Methods why they think they were meaningful and also make this clearer in the Limitations as well.

Please ensure that your revised files adhere to our author guidelines, and that the files are fully copyedited/proofed prior to upload. Please also ensure that all necessary copyright permissions have been obtained. This may be the last opportunity for major editing, therefore please fully check your file prior to re-submission.

If you have any questions or difficulties during this process, please contact the editorial office at [editorialoffice@collabra.org](mailto:editorialoffice@collabra.org).

We hope you can submit your revision within the next six weeks. If you cannot make this deadline, please let us know as early as possible.

Sincerely,
Brenna Hassinger-Das

# Reviewer 1

##### Rating scale questions

|  | Strongly Disagree | Disagree | Neutral | Agree | Strongly Agree |
| --- | --- | --- | --- | --- | --- |
| The study/studies in this manuscript have strong construct validity (good measures and/or manipulations of the constructs the authors wish to study). (Choose “Neutral” if this is not an empirical manuscript) |  |  |  | ✔ |  |
| The study/studies in this manuscript have strong statistical validity (appropriate statistical tests, assumptions are clear and reasonable, no statistical errors, appropriate statistical inferences, etc.). (Choose “Neutral” if this is not an empirical manuscript) |  |  |  | ✔ |  |
| The study/studies in this manuscript have strong internal validity (any causal claims or implications are well-justified, alternative explanations are thoroughly considered, etc.). (Choose “Neutral” if this is not an empirical manuscript, or no causal claims are made or even vaguely implied.) |  |  |  | ✔ |  |
| The study/studies in this manuscript have strong external validity (authors appropriately constrain their conclusions based on the limits of the generalizability of their findings to other contexts (including from lab to real world), other populations, other stimuli or measures, etc.) |  |  | ✔ |  |  |

##### Open response questions

### Please write your review here. The author(s) will see this review. Your identity will not be revealed to the authors unless you also include your name (i.e., sign your review) in this box. It is up to you whether to reveal your identity or not, either is fine.

Overall, the authors have done a good job of addressing my concerns, although the reasons they give for not being able to test more participants aren’t entirely convincing. At the very least, I would urge the authors to address the issue of small sample sizes more clearly in the limitation: “may not have been large enough to detect subtle, age-specific differences within this range” is clearly an understatement of the problem. The current sample sizes are far too small to detect any age-related differences.

I’m still not 100% convinced that the choices the children were able to make in the active condition were (perceived to be) meaningful. Could the authors explain in the Methods why they think they were meaningful? In the Limitations they write: “The nature of the choices and the feedback provided may not have been sufficient to fully engage younger children”. However, the sentences that follow didn’t make it clear to me how the nature of the choices and feedback as implemented in their studies might have been insufficient. Perhaps the authors could make this clearer.

# Reviewer 2

##### Rating scale questions

|  | Strongly Disagree | Disagree | Neutral | Agree | Strongly Agree |
| --- | --- | --- | --- | --- | --- |
| The study/studies in this manuscript have strong construct validity (good measures and/or manipulations of the constructs the authors wish to study). (Choose “Neutral” if this is not an empirical manuscript) |  |  |  | ✔ |  |
| The study/studies in this manuscript have strong statistical validity (appropriate statistical tests, assumptions are clear and reasonable, no statistical errors, appropriate statistical inferences, etc.). (Choose “Neutral” if this is not an empirical manuscript) |  |  |  |  | ✔ |
| The study/studies in this manuscript have strong internal validity (any causal claims or implications are well-justified, alternative explanations are thoroughly considered, etc.). (Choose “Neutral” if this is not an empirical manuscript, or no causal claims are made or even vaguely implied.) |  |  |  | ✔ |  |
| The study/studies in this manuscript have strong external validity (authors appropriately constrain their conclusions based on the limits of the generalizability of their findings to other contexts (including from lab to real world), other populations, other stimuli or measures, etc.) |  |  |  | ✔ |  |

##### Open response questions

### Please write your review here. The author(s) will see this review. Your identity will not be revealed to the authors unless you also include your name (i.e., sign your review) in this box. It is up to you whether to reveal your identity or not, either is fine.

The authors have done a nice job addressing the comments from the previous review, and these changes have in my view substantially improved the manuscript. I have no additional comments at this time.

**Author Response**
Apr 14, 2025

Dear Dr. Hassinger-Das,

Thank you for your thoughtful guidance and for sharing the reviewers’ comments regarding our manuscript, “The Limits of Agency: Young Children’s Memory May Not Benefit from Choice.” We appreciate the time and effort both reviewers invested in evaluating our work. We have carefully addressed each comment and detailed our revisions in the point-by-point responses below. We believe these changes strengthen our manuscript and clarify our rationale regarding sample size considerations, the design’s emphasis on child-friendly immersion, and the role of feedback in our procedures.

Please find our point-by-point responses organized according to each comment. Page numbers refer to the revised manuscript.

1. Clarifying the sample size limitations (Editor + Reviewer 1)
   - We have now revised the Limitations section to more clearly address the issue of sample size, especially regarding age-specific effects. We have added a more explicit discussion about the potential underpowering of any age-by-age comparisons. In other words, once our overall sample is subdivided by individual ages (e.g., 4-year-olds vs. 5-year-olds), the effective sample size per group is substantially reduced, which diminishes our ability to detect more subtle or small effects. Please see page 24 in the revised manuscript for these updates.
     We believe this revision highlights that our overall findings suggest minimal agency benefits for the 4–7 age range, and that further studies with larger, age-stratified samples are needed to ascertain whether small or age-specific agency effects are present.
2. Explaining how children’s choices were intended to be meaningful (Editor + Reviewer 1)
   - In the revised manuscript (see page 11 in the Methods section), we have inserted an additional paragraph clarifying that each choice led to a different set of items and backgrounds, thereby giving the child a tangible sense of influence over the story. We did not explicitly inform children that these elements would change, only that their selection would continue the story—allowing them to discover the consequence of their choice organically. We also added a brief note in the Discussion (see page 23-24) acknowledging that some children may not have noticed how their choices drove these differences, suggesting that future designs could include more explicit feedback to reinforce the causal link between selection and outcome.
3. Addressing the nature of feedback and potential insufficiency (Reviewer 1)
   - In our revision, we clarify in the Methods section (see page 11) that our design intentionally prioritized a child-friendly, immersive experience, allowing children to “help” or make decisions on behalf of the characters (e.g., “You pick one for Bluey!”). This approach, while promoting deeper engagement in the storyline, did not incorporate explicit statements such as “Because you chose X, now Y happens,” which may have reduced the children’s awareness of the causal link between their choices and subsequent scenes. We have now added a paragraph in the Discussion (see page 23-24) explaining that we deliberately avoided “breaking the fourth wall” too frequently to maintain the cartoon’s narrative flow. However, this reduced emphasis on direct feedback could have attenuated any potential agency effect on memory. We suggest that future research compare a more explicit feedback condition (“You chose the yellow door, so you see the living room”) to see whether stronger feedback heightens children’s sense of control and yields greater mnemonic benefits.

**Editor Final Decision:** Accept

Apr 15, 2025

Dear Naoya Tani,

I have now had a chance to read over your manuscript "The Limits of Agency: Young Children’s Memory May Not Benefit from Choice”, along with the letter describing the changes you made. Thank you for your responsiveness to the concerns that the reviewers and I raised. I am happy to say that your paper is now officially accepted for publication in Collabra: Psychology. Congratulations on this excellent work, I think it will make an important contribution to the literature and I look forward to seeing it published! I hope your experiences with Collabra: Psychology have been positive and that you will continue to consider it as an outlet for your work.

As there are no further reviewer revisions to make, you do not have to complete any tasks at this point.

You will be receiving separate correspondence regarding any production and technical comments, data deposits, as well as publication charges. We work with the Copyright Clearance Center to process any applicable APC charges. Please note that your APC transaction must be completed before your article gets published.

You will have an opportunity to check the page proofs before we publish your article. Thank you again for publishing in Collabra: Psychology.

Sincerely,
Brenna Hassinger-Das
Associate Editor
Collabra: Psychology
